# One-step synthesis of Nickle Iron-layered double hydroxide/reduced graphene oxide/carbon nanofibres composite as electrode materials for asymmetric supercapacitor

**DOI:** 10.1038/s41598-018-27171-0

**Published:** 2018-06-11

**Authors:** Feifei Wang, Ting Wang, Shiguo Sun, Yongqian Xu, Ruijin Yu, Hongjuan Li

**Affiliations:** Shaanxi Key Laboratory of Natural Products & Chemical Biology, College of Chemistry & Pharmacy, Northwest A&F University, Xinong Road 22, Yangling, Shaanxi 712100 P.R. China

## Abstract

A novel NiFe-LDH/RGO/CNFs composite was produced by using a facile one-step hydrothermal method as electrode for supercapacitor. Compared with NiFe-LDH/CNFs, NiFe-LDH/CNTs and NiFe-LDH/RGO, NiFe-LDH/RGO/CNFs demonstrated a high specific capacitance of 1330.2 F g^−1^ at 1 A g^−1^ and a super rate capability of 64.2% from 1 to 20 A g^−1^, indicating great potential for supercapacitor application. Additionally, an asymmetric supercapacitor using NiFe-LDH/RGO/CNFs composite as positive electrode material and activated carbon as negative electrode material was assembled. The asymmetric supercapacitor can work in the voltage range of 0–1.57 V. It displayed high energy density of 33.7 W h kg^−1^ at power density of 785.8 W kg^−1^ and excellent cycling stability with 97.1% of the initial capacitance after 2500 cycles at 8 A g^−1^. Two flexible AC//LDH-RGO-CNFs ASC devices connected in series were able to light up a red LED indicator after being fully charged. The results demonstrate that the AC//LDH-RGO-CNFs ASC has a promising potential in commercial application.

## Introduction

Over the past few years, supercapacitor (also known as electrochemical capacitor) has attracted intense interest because of their high energy density, excellent cycling stability, envirinmental friendliness and rapid charge-discharge rate^1–6^. According to the energy storage mechanisms, supercapacitors can generally categorized into electrical double layer capacitor (EDLC) and pseudocapacitor (PC)^7^. It is well known that carbon materials are fundamental candidates for EDLC^8^. Until now, a variety of carbon materials have been used as the electrode materials such as carbon nanotubes (CNTs)^8–10^, carbon nanofibres (CNFs)^11–14^, reduced graphene oxide (RGO) and so on^8,15–17^. Compared with EDLC, PC was major based on transition metal oxides/hydroxides or conducting polymers by redox reactions during charge-discharge processes^8,9,18–20^. It should be noted that, the properties of active electrode materials are the key factors that affect the properties of supercapacitor^21^. Consequently, wonderful electrode materials with high surface area and plenty of activesites are imminently requirement for blossoming electrochemical energy storage and conversion systems^4^.

Layered double hydroxide (LDH), as a family of layered anionic materials, has received considerable attention due to their flexible ion-exchange ability, high specific surface area and flexible structures^5,22,23^. Nevertheless, the relatively low conductivity of LDH often restricts electron transfer and limits the rate of mass diffusion during the redox reaction. Beyond that the agglomeration of LDH may lead to low energy density and limit cycling stability during the charge/discharge processes. As known to all, the electrochemical performances of LDH can be improved effectively when they are combined with carbon materials. As additives, carbon materials can prevent the agglomeration of LDH, improve the specific surface area of the composition and enhance the electrical conductivity of the compound^22,24,25^. To this end, various composites have been producted as high-performance electrode materials of PC. Guan *et al*. reported CoAl-LDH/CNFs can achieve maximum specific capacitance of 634.3 F g^−1^ at 1 A g^−1^ ^26^. Cao *et al*. found that CoNiAl-LDH/RGO (25) exhibited the specific capacitance of 1866 F g^−1^ at 1 A g^−1^. Furthermore, the composite displayed wonderful cycling performances without an obvious capacitance decrease after 5000 cycles^27^. Cheng *et al*. prepared the ternary Ni-Mn LDH/CNTs/RGO composite, and the product yielded the specific capacitance of 1268 F g^−1^ in 2 M KOH electrolyte^28^. Our previous work found that NiFe-LDH material with high crystallinity and well-defined hexagonal shapes was perfect candidate for energy store^29^. However, NiFe-LDH material has always been investigated for water splitting or water oxidation but not often has been evaluated its electrochemical properties for supercapacitor application^23,30^.

In this work, we synthesised NiFe-LDH/CNFs, NiFe-LDH/CNTs, NiFe-LDH/RGO and NiFe-LDH/RGO/CNFs composites for supercapacitor electordes by a hydrothermal route under mild conditions. Compared with other composites, the NiFe-LDH/RGO/CNFs composite expressed the best electrochemical performance. The composite demonstrated a high specific capacitance of 1330.2 F g^−1^ at 1 A g^−1^ and a super rate capability of 64.2% from 1 to 20 A g^−1^, indicating great potential for supercapacitor applications. Moreover, an asymmetric supercapacitor using NiFe-LDH/RGO/CNFs composite as positive electrode material and AC as negative electrode material was assembled. The asymmetric supercapacitor showed large energy density of 33.7 W h kg^−1^ at power density of 785.8 W kg^−1^ and wonderful cycling stablity with 97.1% of the initial specific capacitance after 2500 test cycles at 8 A g^−1^, manifesting its promising application in the future.

## Results and Discussion

**Figure 1:**
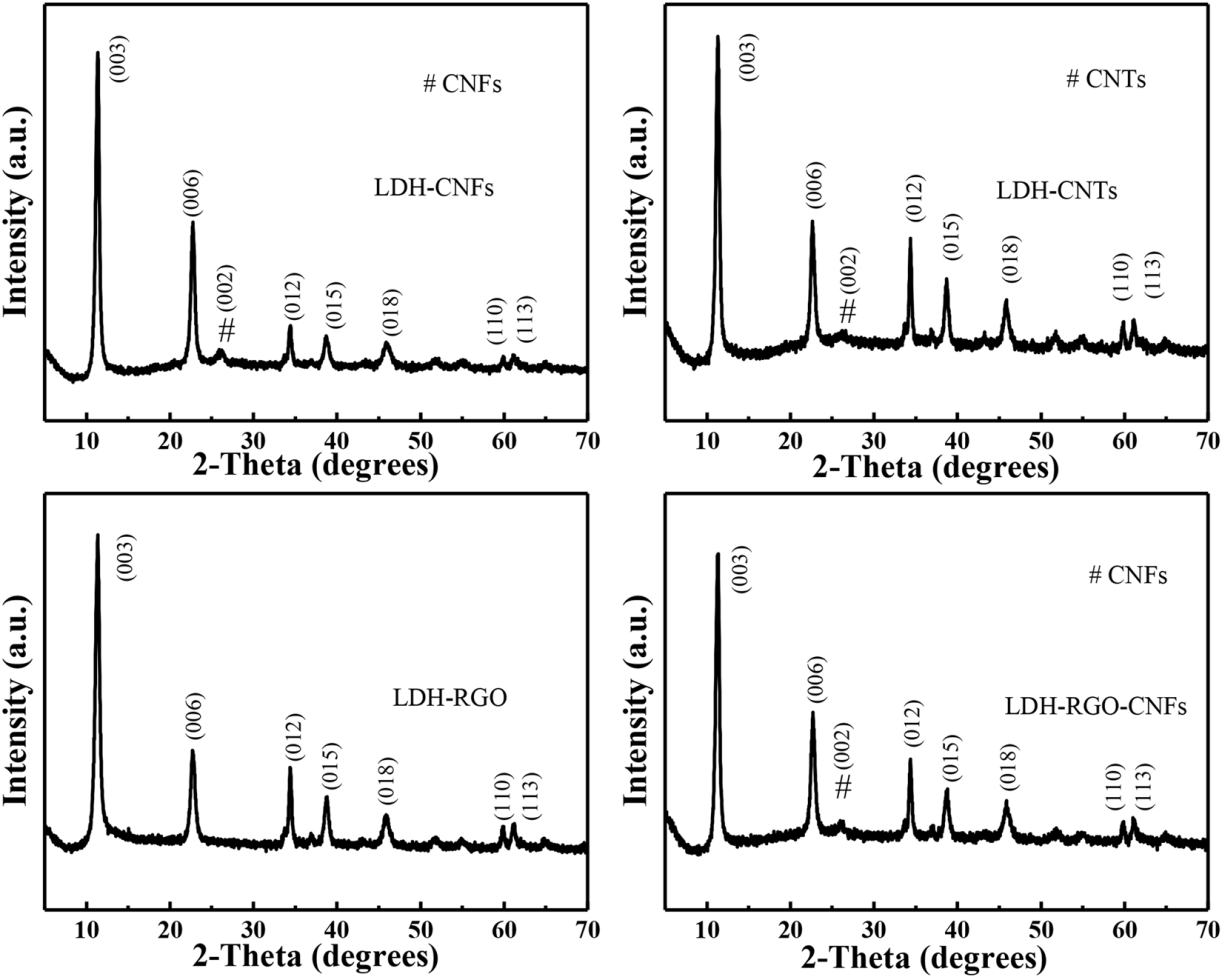
XRD spectra of LDH-CNFs, LDH-CNTs, LDH-RGO and LDH-RGO-CNFs.

X-ray diffraction (XRD) is conducted to study the structure of the as-synthesized materials. The XRD patterns of LDH-CNFs, LDH-CNTs, LDH-RGO and LDH-RGO-CNFs are shown in Fig. 1. The diffraction peaks at 11.3°, 22.6°, 34.4°, 38.7°, 45.8°, 59.9°, and 61.0° can be assigned to the (003), (006), (012), (015), (018), (110) and (113) planes of the hydrotalcite-like LDH phase. Furthermore, for LDH-CNFs, LDH-CNTs and LDH-RGO-CNFs, the diffraction peak at 24.5° belong to (002) plane of graphite, demonstrating a successful production of LDH-CNFs, LDH-CNTs and LDH-RGO-CNFs^27,31^. The absence of the peak of RGO in LDH-RGO manifested that the RGO nanosheets were tightly embedded by LDH nanosheets^24,31^.

Moreover, the Raman spectra of CNFs, GO and LDH-RGO-CNFs are shown in Fig. S1. Two major peaks at 1350 and 1580 cm^−1^ belonged to D and G bands of carbon materials, respectively. D band reflected the defect and the disordered structure of carbon, and G band was attributed to the graphitic in-plane vibrations of ideal sp^2^ carbons^31^. In addition, two absorption peaks at 569.6 and 670.2 cm^−1^ were apparent in the Raman spectrum of LDH-RGO-CNFs, which should be the feature peaks of NiFe-LDH, indicating the coexistence of carbon material and LDH^32,33^.

**Figure 2:**
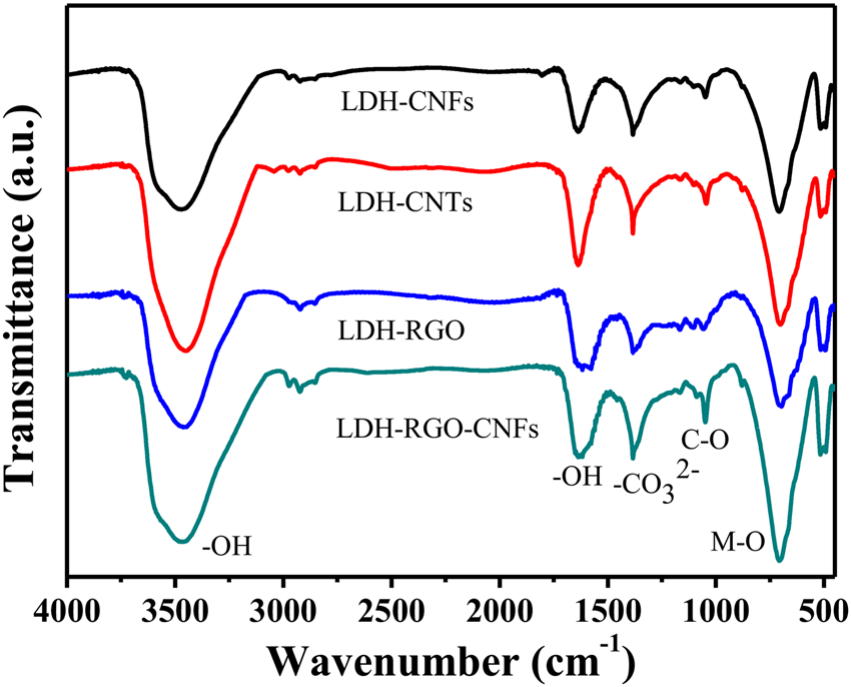
FT-IR spectra of LDH-CNFs, LDH-CNTs, LDH-RGO and LDH-RGO-CNFs.

The chemical bonding nature of the LDH-CNFs, LDH-CNTs, LDH-RGO and LDH-RGO-CNFs was tested by FT-IR spectroscopy, as shown in Fig. 2. The infrared band around 3460 cm^−1^ can be assigned to the OH-stretching vibration in the brucite-like layers and interlayer water, accompanied by the band at 1633 cm^−1^ ^28^. In addition, the band at 1383 cm^−1^ was ascribed to the vibration of CO_3_^2−^ ^34^. It can be observed that the stretching vibrations of C-O was at 1049 cm^−1^ ^35,36^. Other absorption bands below 800 cm^−1^ were assigned to metal-oxygen (M-O) stretching and bending vibration in the brucite-like lattice^36^, confirming the existence of NiFe-LDH in the composites.

**Figure 3:**
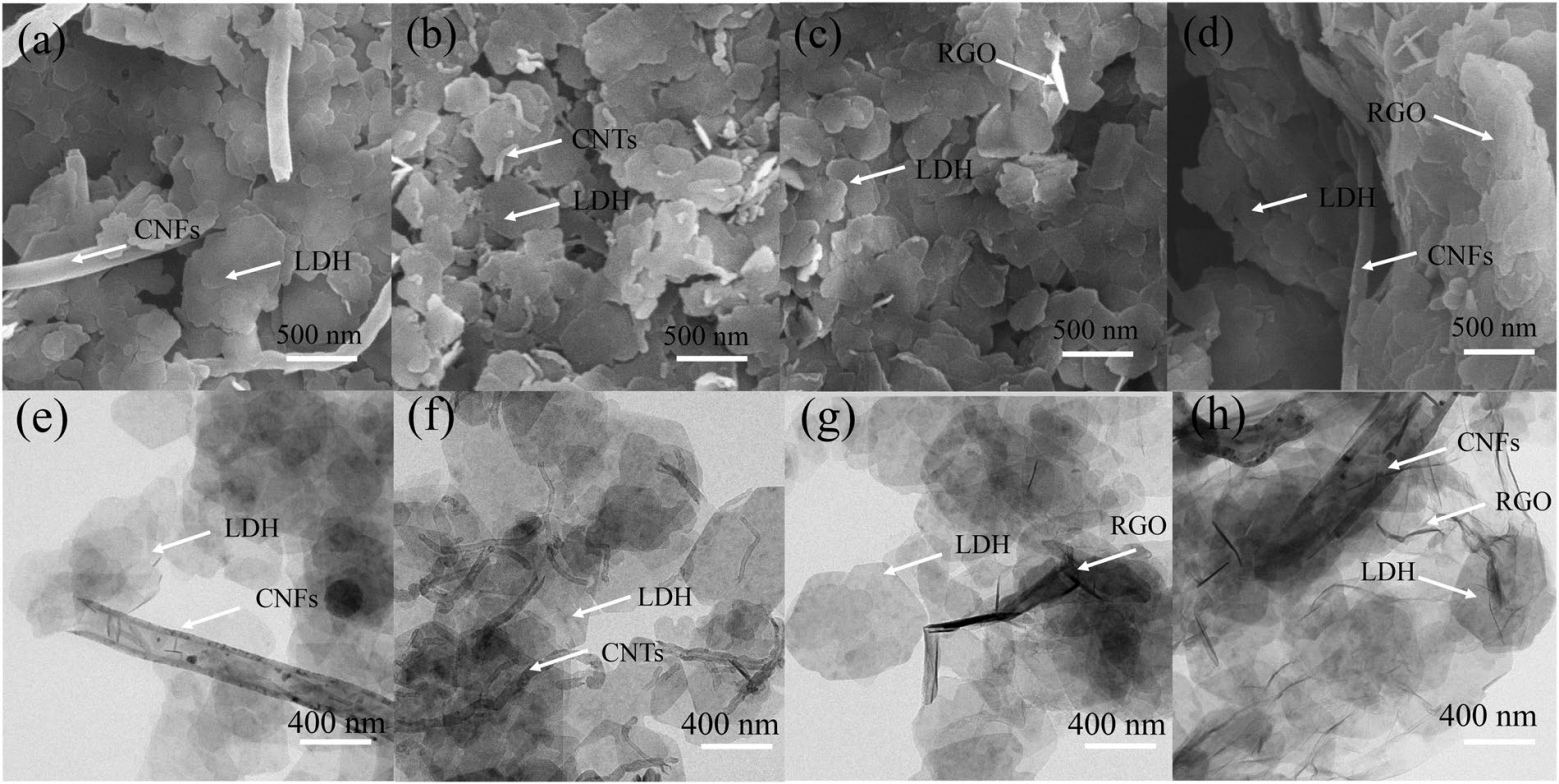
FESEM images of (**a**) LDH-CNFs, (**b**) LDH-CNTs, (**c**) LDH-RGO and (**d**) LDH-RGO-CNFs. TEM images of (**e**) LDH-CNFs, (**f**) LDH-CNTs, (**g**) LDH-RGO and (**h**) LDH-RGO-CNFs.

The morphology and structure of the LDH-CNFs, LDH-CNTs, LDH-RGO and LDH-RGO-CNFs were characterized by FESEM and TEM. As shown in Fig. 3a, the NiFe-LDH displayed the irregular nanoplates and CNFs intercrossed with each other. Therefore, the aggregation of LDH was effectively avoided by introducing CNFs into LDH. From the TEM images of LDH-CNFs (Fig. 3e), the CNFs exhibited a hollow tubular structure with the inner diameter about 100 nm. From Fig. 3b, we can see that the LDH-CNTs was composed of numerous LDH nanosheets and CNTs randomly dispersed on the surfaces of LDH platelets. Compared with CNFs, CNTs (Fig. 3f) showed a smaller diameter, which was not conducive to prevent the aggregation of LDH. As shown in Fig. 3c and g, the LDH nanosheets were disorderly entangled with scrolled and corrugated RGO nanosheets. As expected, the LDH-RGO-CNFs composite (Fig. 3d and h) combined the morphologies of both LDH-CNFs and LDH-RGO. Furthermore, the loose structure of LDH-RGO-CNFs may provide numerous electrochemically active sites and lead to higher specific capacitance. In addition, the incorporation of RGO into LDH can prevent LDH nanosheets from aggregation and improve the specific surface area of the composite. Meanwhile, the CNFs can enhance the electrical conductivity of nanohybrids as a conducting scaffold. The EDS spectra of the LDH-CNFs, LDH-CNTs, LDH-RGO and LDH-RGO-CNFs composites are presented in Fig. S2. The elements of C, O, Ni, Fe, Pt, and Si were detected. The Pt signal came from the plated element for SEM measurement. The peak of Si originated from the silicon slice.

**Figure 4:**
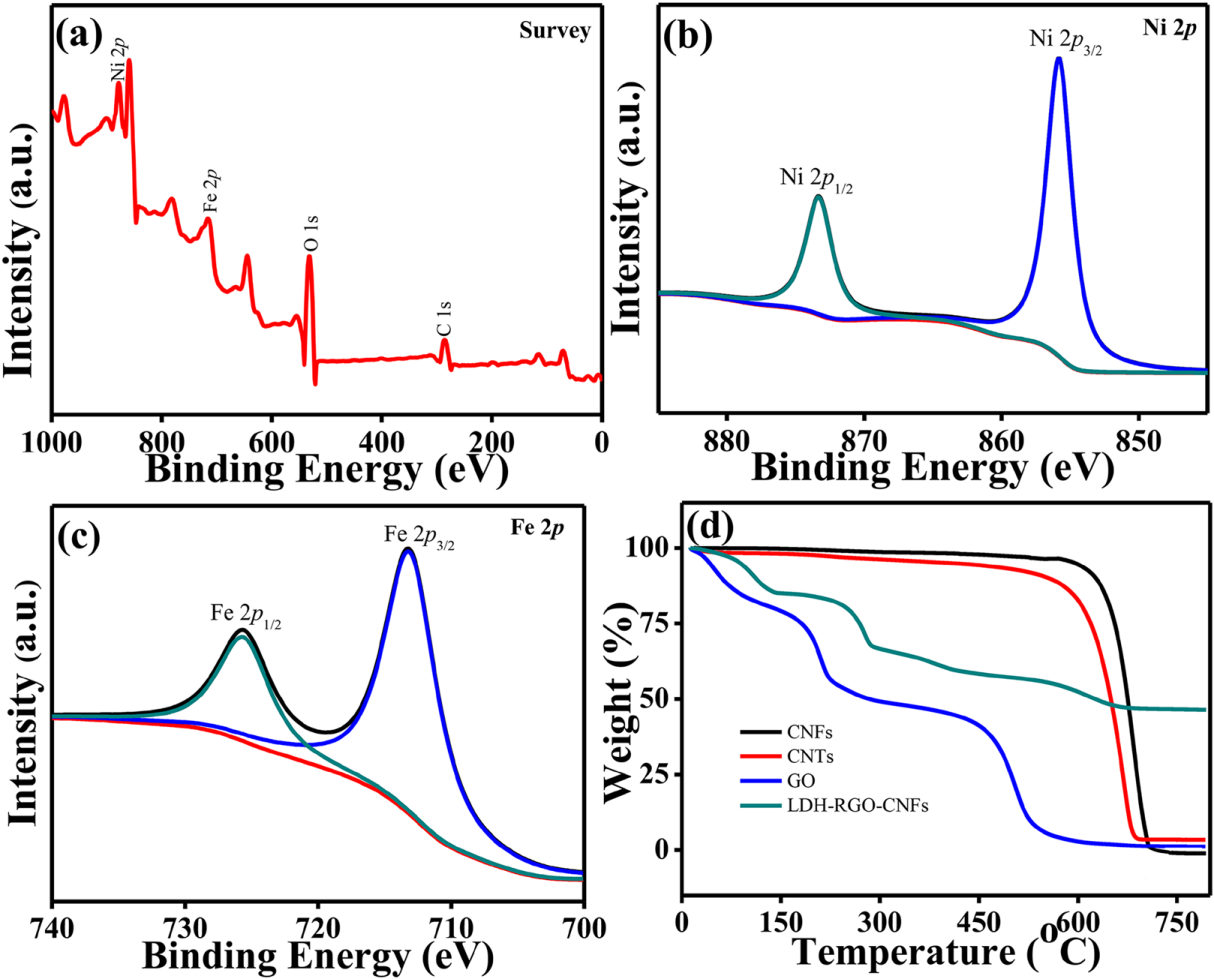
XPS survey and high-resolution spectra of LDH-RGO-CNFs. (**a**) Survey, (**b**) Ni 2*p*, (**c**) Fe 2*p*; (**d**) TGA curves of CNFs, CNTs, GO, LDH-RGO-CNFs.

Furthermore, X-ray photoelectron spectroscopy (XPS) was also performed for CNFs, CNTs, GO, and LDH-RGO-CNFs, as shown in Fig. S3 and Fig. 4. Characteristic peaks for C, Ni, Fe and O elements were indicated in the survey spectrum (Fig. 4a). In Fig. 4b, peaks at the binding energy of 855.8 eV and 873.4 eV were related to Ni 2*p*_3/2_ and Ni 2*p*_1/2_, respectively. Moreover, the spin orbit splitting value of Ni 2*p*_3/2_ and Ni 2*p*_1/2_ can reach 17.6 eV, indicating that the main oxidation state of Ni is +2^31^. The Fe 2*p* (Fig. 4c) spectrum showed prominent peaks at 713.9 eV for Fe 2*p*_3/2_ and 725.5 eV for Fe 2*p*_1/2_^37^. As shown in Fig. S3, compared with the C1s spectrum of GO, the intensities of oxygen-containing functional groups were decreased in LDH-RGO-CNFs, confirming the well reduction of GO into RGO^38,39^.

The thermal stability of CNFs, CNTs, GO, and LDH-RGO-CNFs was researched by thermogravimetric analysis (TGA). As shown in Fig. 4d, three significant weight loss stages were observed in the TGA pattern of the LDH-RGO-CNFs. The first weight loss was due to the removal of surface and intercalated water molecules^22^. The second weight loss was the removal of interlayer anions and the de-hydroxylation of LDH crystals^17,40^. The third weight loss was assigned to the the combustion of the carbon skeleton^40^. The residue percentage of the LDH-RGO-CNFs was much higher than CNFs and GO, indicating that the combination of LDH with carbon materials significantly improved the thermal stability.

The electrochemical behavior of LDH-RGO_X_-CNFs (X = 0.25, 0.5, 1, 2, 4) composites with different mass ratios of RGO and CNFs were studied by cyclic voltammetry (CV) and galvanostatic charge-discharge (GCD). As shown in Fig. S4, the specific capacitance of LDH-RGO-CNFs (1330.2 F g^−1^) was superior than LDH-RGO_0.25_-CNFs (601.4 F g^−1^), LDH-RGO_0.5_-CNFs (642.1 F g^−1^), LDH-RGO_2_-CNFs (743.2 F g^−1^) and LDH-RGO_4_-CNFs (661.1 F g^−1^) at 1 A g^−1^. Thus, the LDH-RGO-CNFs was deemed as the best suitable sample for supercapacitor electrode.

**Figure 5:**
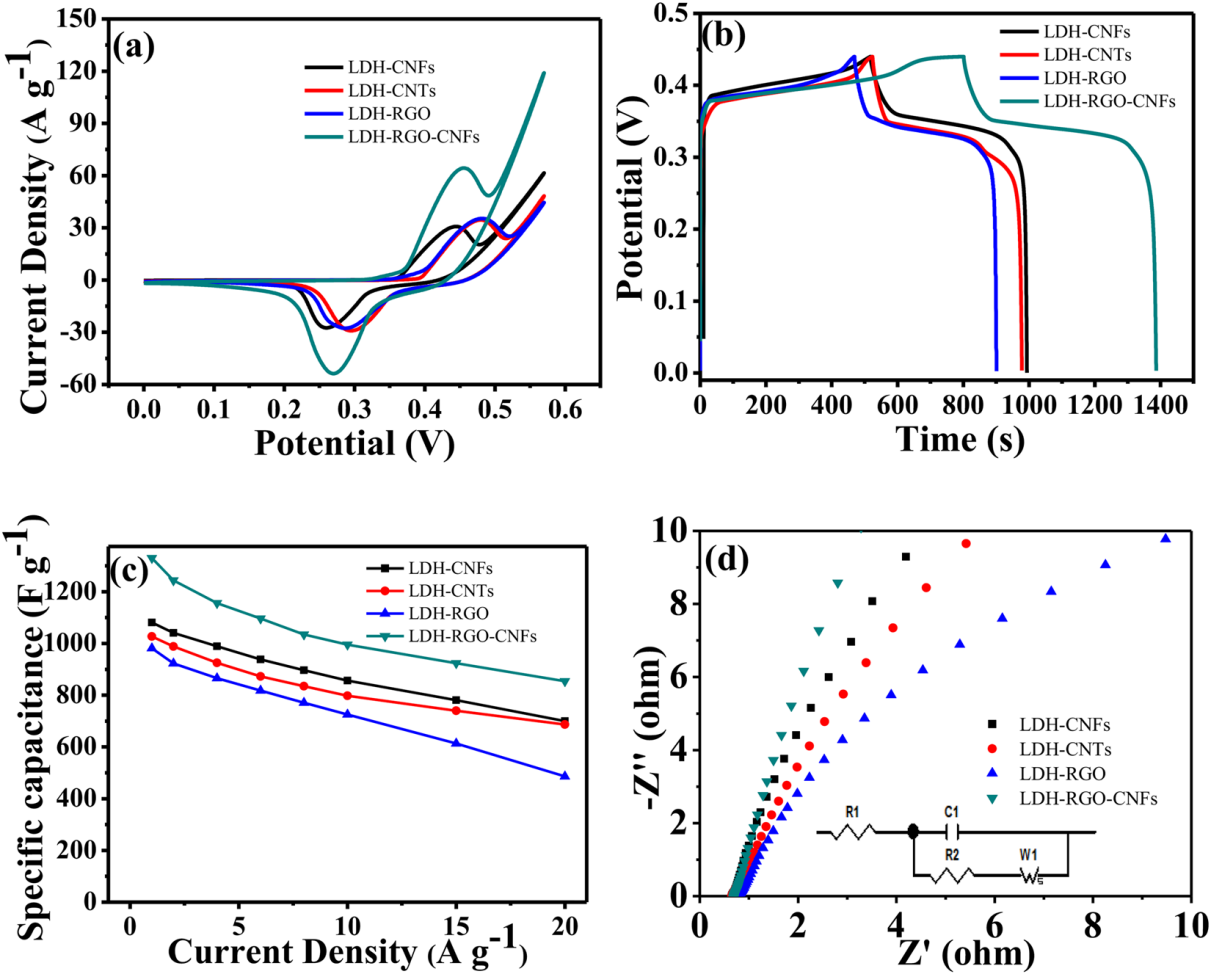
Electrochemical capacitive properties of LDH-CNFs, LDH-CNTs, LDH-RGO and LDH-RGO-CNFs composites tested using a three-electrode system in 6 M KOH solution. (**a**) CV curves at 10 mV s^−1^. (**b**) GCD curves at 1 A g^−1^. (**c**) The specific capacitance at different current densities. (**d**) The Nyquist plots measured at frequency range between 0.01 and 10^5^ Hz. Inset: simplified equivalent circuit.

In order to evaluate the electrochemical performances of the LDH-CNFs, LDH-CNTs, LDH-RGO and LDH-RGO-CNFs composites, the samples were studied by CV, GCD and EIS. Figure 5a shows the CV curves of the four samples in the 6 M KOH at 10 mV s^−1^ in a voltage range of 0–0.57 V. All CV curves consisted of a couple of redox peaks, manifesting that the capacitance of the products was mainly associated with faradaic pseudocapacitor. In addition, compared with the LDH-CNFs, LDH-CNTs and LDH-RGO, the LDH-RGO-CNFs exhibited a largest integrated area, indicating the LDH-RGO-CNFs had highest specific capacitance^31^. Figure S5 shows the CV curves of the LDH-CNFs, LDH-CNTs, LDH-RGO and LDH-RGO-CNFs composites at different scan rates. With the increased of the scan rate, the current response also increased without any obvious changes in the shape of the CV, meaning a good rate capability of the active materials^41,42^. Furthermore, the anodic peaks shifted positively and cathodic peaks shifted negatively, respectively, which was mainly due to the internal resistance of electrode^8,43^.

To further illustrate the electrochemical performances of the composites, GCD measurements were conducted. Figure 5b displays the charge/discharge curves of the LDH-CNFs, LDH-CNTs, LDH-RGO and LDH-RGO-CNFs composites at 1 A g^−1^ in a voltage range of 0–0.44 V. It is clearly seen that the LDH-RGO-CNFs composite demonstrated the longest charge/discharge time, implying the highest specific capacitance. The charge/discharge curves of the LDH-CNFs, LDH-CNTs, LDH-RGO and LDH-RGO-CNFs composites at different current densities are shown in Fig. S6. It showed that the discharge times decreased with the increase of the current density from 1 to 20 A g^−1^. It can be attributed to the limited diffusion rate of the alkali ions. When the current densities increased, the diffusion rate of the alkali ions becomed relatively low, and thus only the outer active surface of the electrode material can be utilized for charge storage during the redox process^17^. Furthermore, no apparent IR drop was observed in the curves of LDH-RGO-CNFs, meaning that the composite had a low internal resistance. The specific capacitances obtained from GCD tests can be calculated according to eq. (1), as shown in Fig. 5c and Table S1. The outstanding specific capacitances of LDH-RGO-CNFs at 1, 2, 4, 6, 8, 10, 15, 20 A g^−1^ were 1330.2, 1244.1, 1155.5, 1096.4, 1034.5, 995.5, 923.9 and 854.5 F g^−1^, respectively. The retention rate of LDH-RGO-CNFs (64.2%) was larger than those of LDH-RGO (49.5%), LDH-CNTs (63.2%) and LDH-CNFs (63.9%) from 1 to 20 A g^−1^. (Table S1) It can be explained that the introduction of RGO was effectively avoided the aggregation of LDH and prevented the loss of the active surface area of the composite. In addition, adding CNFs not only can effectively increase the electrical conductivity of the composite, but also can improve the spacing of graphene layer, leading to a fast electrolyte ion diffusion path during the charge/discharge processed^42^. Therefore, the electrochemical performances of the LDH-RGO-CNFs composite have been improved.

The electrochemical impedance technique (EIS) was used to investigate the electrochemical performances of the LDH-CNFs, LDH-CNTs, LDH-RGO and LDH-RGO-CNFs composites in a frequency range between 0.01 and 10^5^ Hz. The Nyquist plots of the EIS recorded for the composites are shown in Fig. 5d, and the inset shows the simplified equivalent circuit. R_e_ represented the resistance related to the ionic conductivity of the electrolyte and electronic conductivity of the electrodes and current collectors^43^, which can be valued by the intercept of a quasi-semicircle in the x axis^42^. The semicircle in the high frequency region represents the charge transfer resistance (R_ct_) at the electrode/electrolyte interface^9^. Evidently, in high frequency area, the R_e_ of LDH-RGO-CNFs exhibited a small x axis intercept and a negligible semicircle, indicating a very low internal resistance and charge transfer resistance. Besides, compared with LDH-CNFs, LDH-CNTs and LDH-RGO, LDH-RGO-CNFs had a more vertical line in the low frequency area, confirming a lower Warburg impedance^32^. These results demonstrated that the addition of CNFs and RGO improved the conductivity of the product and decreased the impedance.

**Figure 6:**
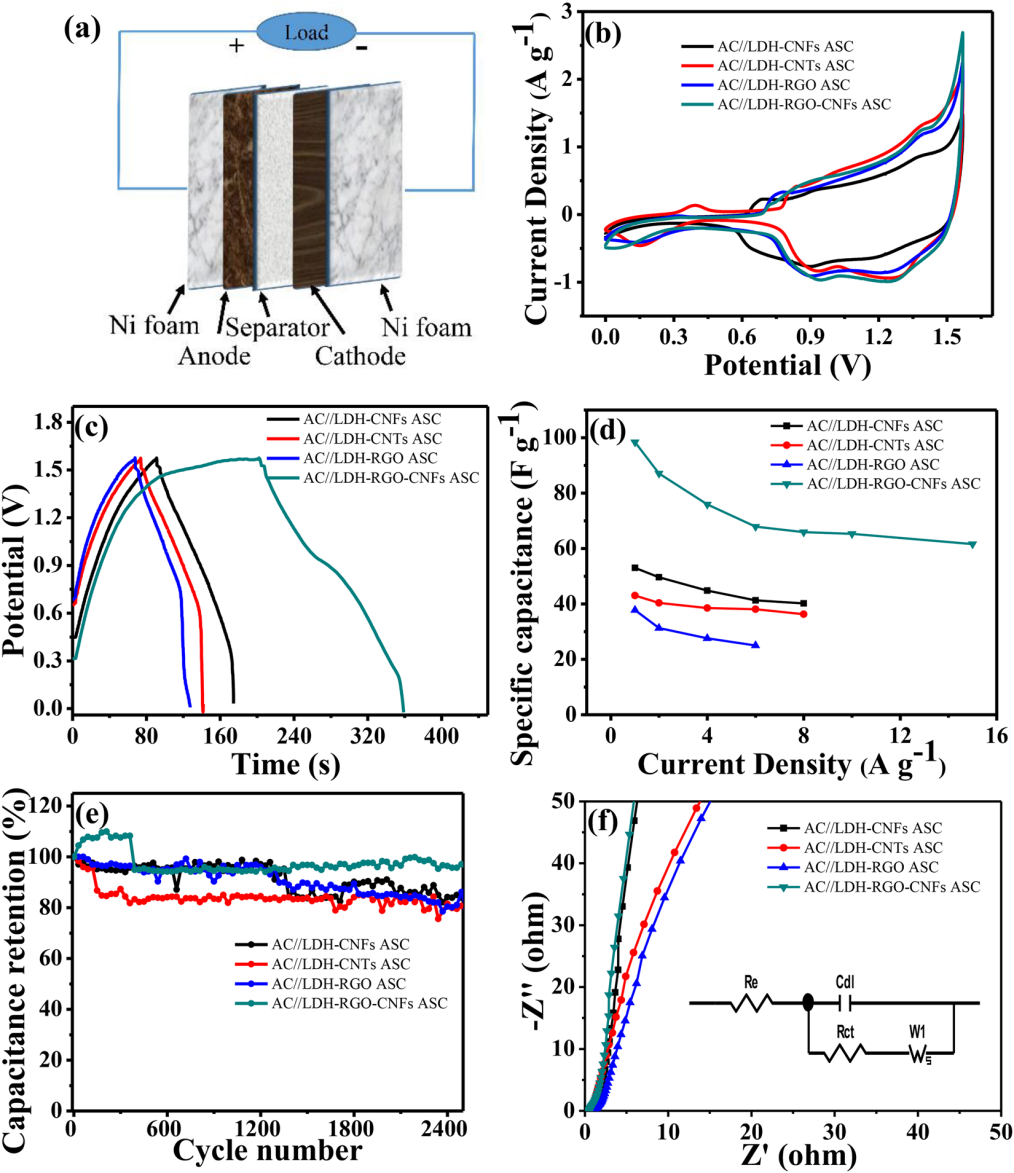
(**a**) The schematic of the ASC. Electrochemical capacitive properties of AC//LDH-CNFs ASC, AC//LDH-CNTs ASC, AC//LDH-RGO ASC and AC//LDH-RGO-CNFs ASC. (**b**) CV curves at 10 mV s^−1^. (**c**) GCD curves at 1 A g^−1^. (**d**) The specific capacitance at different current densities. (**e**) The specific capacitance retention with 2500 cycling numbers for the AC//LDH-CNFs ASC, AC//LDH-CNTs ASC and AC//LDH-RGO-CNFs ASC at 8 A g^−1^ and AC//LDH-RGO ASC at 6 A g^−1^. (**f**) The Nyquist plots measured in a frequency range between 0.01 and 10^5^ Hz. Inset: simplified equivalent circuit.

To further evaluate the electrochemical properties of the as-prepared composites for pratical application, ASCs were assembled by using the as-prepared composites as the positive electrode material and AC as the negative electrode material (Fig. 6a). Figure S7a shows the CV curves of AC and LDH-RGO-CNFs electrodes at 10 mV s^−1^. The AC and LDH-RGO-CNFs electrodes were operated at voltage windows of −1.0–0 V and 0–0.57 V, respectively. Therefore, the total cell voltage of the ASC device can be increased up to 1.57 V, which was the sum of the voltage range for the AC and LDH-RGO-CNFs electrodes^44^. Figure S7b displays the CV curves of the ASC working at different cell voltages varying from 0–1 V to 0–1.8 V at 10 mV s^−1^. The CV curves were stable at 1.57 V. However, when the voltage was higher than 1.57 V, the CV curves exhibited a distortion which can be attributed to some irreversible reactions occurring. Therefore, an operation voltage range of 1.57 V was chosen to investigate the electrochemical performances of ASCs. Figure 6b and c shows the CV and GCD curves of AC//LDH-CNFs ASC, AC//LDH-CNTs ASC, AC//LDH-RGO ASC and AC//LDH-RGO-CNFs ASC in the voltage range of 0–1.57 V. Clearly, the AC//LDH-RGO ASC exhibited the largest enclosed area of CV curves and longest discharge time of GCD curves, indicating its superior capacitance performance. In addition, Figs S8 and S9 shows the CV and GCD curves of four ASCs at different scan rates and current densities, respectively. The results were consistent with the CV and GCD analysis in the three-electrode configuration. Figure 6d and Table S2 displays the specific capacitance of the four ASCs at different current densities. The AC//LDH-RGO-CNFs ASC demonstrated the specific capacitance of 98.4, 87.1, 75.9, 67.9, 65.9, 65.3 and 61.6 F g^−1^ at 1, 2, 4, 6, 8, 10 and 15 A g^−1^, which were higher than those of the other three ASCs at the same current density. In addition, The long-term cycling stability of the four ASCs were tested and the result is shown in Fig. 6e. The capacity of AC//LDH-RGO-CNFs ASC can retain 97.1% of its initial capacity after 2500 consecutive GCD tests at 8 A g^−1^, which was better than the capacitance retention of AC//LDH-CNFs ASC (83.9% retention at 8 A g^−1^), AC//LDH-CNTs ASC (80.7% retention at 8 A g^−1^) and AC//LDH-RGO ASC (86.2% retention at 6 A g^−1^). Initial increase of the specific capacitance of the AC//LDH-RGO-CNFs ASC showed an excellent cycling stability at high current density. EIS of the ASCs were measured in a frequency range between 0.01 and 10^5^ Hz (Fig. 6f). The inset of Fig. 6f shows the simplified equivalent circuit. The R_ct_ of AC//LDH-RGO-CNFs ASC (0.6 Ω) was lower than AC//LDH-CNFs ASC (0.65 Ω), AC//LDH-CNTs ASC (0.73 Ω) and AC//LDH-RGO ASC (1.36 Ω), indicating good charge-transfer conductivity of the device at the electrolyte-electrode interface. Otherwise, AC//LDH-RGO-CNFs ASC exhibited the straight line close to 90° in the low frequency, suggesting a lower Warburg impedance.

**Figure 7:**
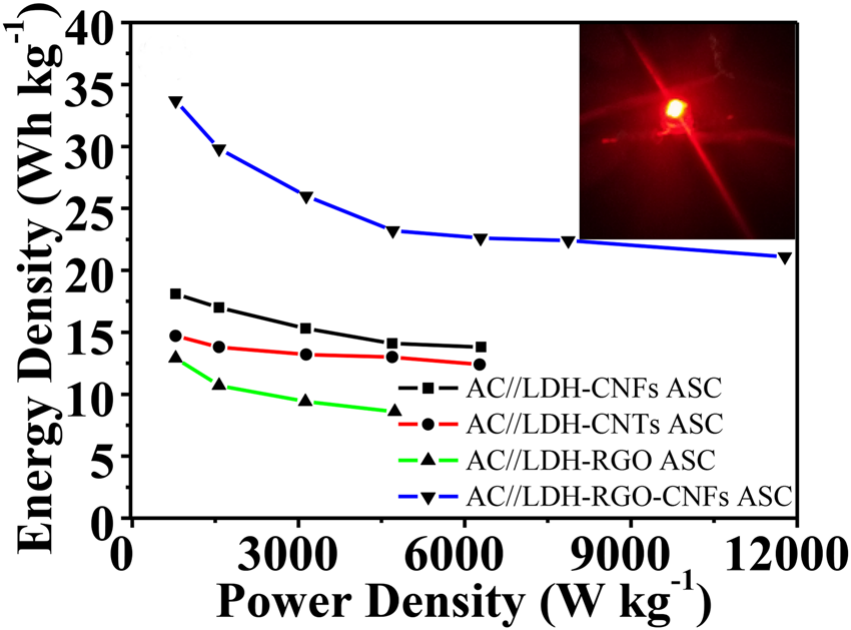
Ragone plot of the energy density vs. power density for the ASCs. The inset shows that two AC//LDH-RGO-CNFs ASC devices in series lighting up a red LED indicator.

Energy density and power density have been widely used to evaluate the performance of the ASCs. According to eqs (3) and (4), the energy density and power density were calculated and shown in Fig. 7. Obviously, the AC//LDH-RGO-CNFs ASC exhibited much higher energy density than the other three ASCs. The maxmum energy density of the AC//LDH-RGO-CNFs ASC was 33.7 W h Kg^−1^ at the power density of 785.8 W Kg^−1^, and still remained at 21.1 W h Kg^−1^ at a power density of 11776.6 W Kg^−1^. The AC//LDH-RGO-CNFs ASC device displayed improved energy density and power density in aqueous electrolyte solutions compared with many previous reported asymmetric supercapacitors, such as AC//CoMn-LDH /Ni foam ASC (5.9 W h Kg^−1^)^45^, AC//MnO_2_/reclaimed CNFs ASC (22.9 W h Kg^−1^)^46^, AC/CFP//Co(OH)_2_/GNS ASC (19.3 W h Kg^−1^)^47^, AC//NiCo-DH ASC (17.5 W h Kg^−1^)^48^, AC//NiCo-LDH ASC (23.7 W h Kg^−1^)^49^, AEG//CoAl-LDH/GF ASC (28.0 W h Kg^−1^)^36^ and AC//Co_3_O_4_-CHCF ASC (24.3 W h Kg^−1^)^50^. It proved that production of LDH-RGO-CNFs composite could be an effective approach in achieving high energy density while retaining power density. Two pieces of flexible AC//LDH-RGO-CNFs ASC devices connected in series were able to light up a red LED indicator (inset in Fig. 7). These results further proved the superiorities of the AC//LDH-RGO-CNFs ASC devices and their great promise for flexible energy storage applications.

## Experimental

### Materials

All the chemicals were used as received. CNFs were obtained from Sigma-Aldrich. Natural flake graphite (325 mesh) was purchased from Alfa-Aesar Co. CNTs with a diameter of 10–20 nm were purchased from Shenzhen Nanotech Port Co. Ltd.

### Surface modification of CNFs and CNTs and preparation of GO

GO was producted by modified Hummers method as reported by our previous work^51^. To increased the dispersability in water, the introduction of surficial groups onto the surface of CNFs were carried out according to ref.^11^. The purchased CNFs were stirred in 68% HNO_3_ with sonication for 30 min and refluxed at 100 °C overnight. Then, the acid-treated CNFs were washed with deionised water to neutral pH and dried at 80 °C overnight. The negatively charged CNTs was prepared according to the same method.

### Preparation of the NiFe-LDH/carbon composites

The NiFe-LDH/carbon composites were produced by an easy *in situ* growth approach. Foremost, 40 mg of carbon materials (CNFs, CNTs, GO and a mixture of GO and CNFs with the mass ratio of 1:4, 1:2, 1:1, 2:1 and 4:1) were dispersed in 80 mL of deionised water with ultrasonication for 60 min. Then, Ni(NO_3_)_2_·6H_2_O (0.4653 g) and Fe(NO_3_)_3_·9H_2_O (0.1616 g) were dissolved in the above homogeneous slurry. After ultrasonication for 20 min in air at room temperature, CO(NH_2_)_2_ (0.2162 g) and Na_3_C_6_H_5_O_7_ (0.0060 g) were added under constant magnetic stirring. Afterwards, the resulted mixture was transferred into a Teflon-lion stainless steel autoclave and kept at 150 °C for 48 h. After the autoclave was cooled down to room temperature, the collected sample was obtained by vacuum filtration, rinsed with deionised water and alcohol for neutralization and dried at 80 °C overnight. The resultant samples were named as LDH-CNFs, LDH-CNTs, LDH-RGO, LDH-RGO_X_-CNFs (X = 0.25, 0.5, 1, 2, 4).

### Material characterization

The crystalline structures of the samples were analyzed by X-ray diffraction (XRD, D/Max2550VB+/PC X-ray, Cu Kα, 0.15406 nm). FESEM (Zeiss Merlin) and TEM (JEM-2100) was used to observe the microstructures of the material. Fourier transform infrared (FT-IR) spectra was obtained on a Brucher EQUINX55 FT-IR spectrophotometer by a standard KBr disk method in the range 400–4000 cm^–1^. Chemical valence states and elemental composition of the samples were analysed by X-ray photoelectron spectroscopy (XPS, Escalab 250Xi, UA). Raman spectrum was performed on a RENISHAW. Thermogravimetric analysis (TGA, NETZSCH, STA 449 F3) was performed in a temperature range from 20 to 800 °C with the heating rate of 10 °C min^−1^ in air.

### Preparation of the electrodes

The obtained sample, acetylene black and polyvinylidene fluoride with a weight ratio of 80:15:5 were mixed and dispersed in ethanol to form homogeneous slurries^15^. Then, the slurries was coated onto a piece of Ni foam and dried at 80 °C overnight to form the working electrode.The resulting electrode was pressed at a pressure of 10 MPa before use.

### Electrochemical measurements

The electrochemical tests of various samples were conducted using a three electrode system in a solution of 6 M KOH with electrochemical workstation (CHI 660E, Chenhua, Shanghai). Hg/HgO electrode and Pt plate were used as the reference electrode and counter electrode, respectively. A salt-bridge was used to connect Hg/HgO electrode and electrolyte. Cyclic voltammetry (CV) in the range of 0–0.57 V was performed at various scan rates. Galvanostatic charge/discharge (GCD) were recorded in the potential range of 0–0.44 V at different current densities. Electrochemical impedance spectroscopy (EIS) was tested in the frequency range between 0.01 and 10^5^ Hz at the open circuit voltage with an alternating amplitude of 5 mV. Cycle-life test was measured by a battery test system (LAND CT2001A). Asymmetric supercapacitors (ASCs) were produced with NiFe-LDH/carbon materials and activated carbon (AC) as positive electrode and negative electrode, respectively. The electrolyte was 6 M KOH. The negative electrode was fabricated by the similar procedure. The specific capacitance of a single electrode was calculated from the charge/discharge curve according to the following equations^15^:

1 $${C}_{S}=\frac{I\times t}{{\rm{\Delta }}V\times m}$$

herein, C_s_ (F g^−1^) is specific capacitance, I (A) is the discharge current, Δt (s) is the discharge time, m (g) is the mass of the sample coated on the Ni foam surface and ΔV (V) is the discharge voltage range.

To achieve optimal electrochemical performance, the mass ratio of positive and negative electrode is received according to eq. (2):

2 $$\frac{{m}_{+}}{{m}_{-}}=\frac{{C}_{-}\times {\rm{\Delta }}{V}_{-}}{{C}_{+}\times {\rm{\Delta }}{V}_{+}}$$

herein, m (g) is the mass loading, C (F g^−1^) is the specific capacitance, ΔV (V) is the discharge voltage range for the positive (+) and negative (−) electrodes.

The energy density and power density of the ASCs were calculated according to eqs (3) and (4), respectively:

3 $$E=\frac{1}{2}\mathrm{Cs}{\rm{\Delta }}{V}^{2}$$

4 $$P=\frac{E\times 3600}{t}$$

herein, E (W h kg^−1^) is energy density, P (W h kg^−1^) is power density, C_s_ (F g^−1^) is specific capacitance, ΔV (V) is the discharge voltage range and Δt (s) is the discharge time.

## Summary

In summary, we have successfully synthesized the LDH-CNFs, LDH-CNTs, LDH-RGO and LDH/RGO/CNFs composites with a facile one-step hydrothermal method, and studied their electrochemical properties in three and two electrode systems. The LDH/RGO/CNFs composite exhibited the highest specific capacitance of 1330.2 F g^−1^ at 1 A g^−1^ in three electrode systems. An asymmetric supercapacitor was fabricated by the LDH/RGO/CNFs composite as positive electrode and AC as negative electrode. The AC//LDH-RGO-CNFs ASC could operate at a wide voltage range of 0–1.57 V, delivered a large energy density of 33.7 W h kg^−1^ at power density of 785.8 W kg^−1^ and showed a remarkable cycling stability with 97.1% of the initial capacitance after 2500 cycles at 8 A g^−1^. Notably, two flexible AC//LDH-RGO-CNFs ASC devices connected in series were able to light up a red LED indicator after being fully charged. The remarkable electrochemical performances indicated that the LDH-RGO-CNFs composite was a promising electrode material and had great application potential for energy storage.

## Electronic supplementary material

Supplementary Information
