# Supplementary material for: One-step synthesis of Nickle Iron-layered double hydroxide/reduced graphene oxide/carbon nanofibres composite as electrode materials for asymmetric supercapacitor
Source: Sci Rep. 2018 Jun 11;8:8908. doi: 10.1038/s41598-018-27171-0 (PMC5995954; doi:10.1038/s41598-018-27171-0)
Supplement: Supplementary file 1 — Supplementary Information [file 41598_2018_27171_MOESM1_ESM.pdf]

Supplementary Information

One-Step synthesis of Nickle Iron-layered double hydroxide/reduced graphene oxide/carbon nanofibres composite as electrode materials for asymmetric supercapacitor

Feifei Wang, Ting Wang, Shiguo Sun, Yongqian Xu, Ruijin Yu and Hongjuan Li*

Shaanxi Key Laboratory of Natural Products & Chemical Biology, College of Chemistry & Pharmacy, Northwest A&F University, Xinong Road 22, Yangling, Shaanxi 712100, P. R. China

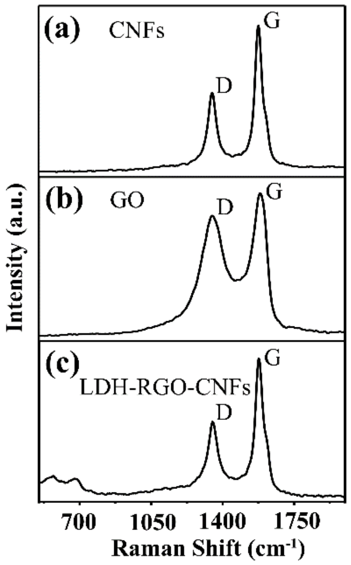

Fig S1 Raman spectrum of (a) CNFs, (b) GO and (c) LDH-RGO-CNFs.

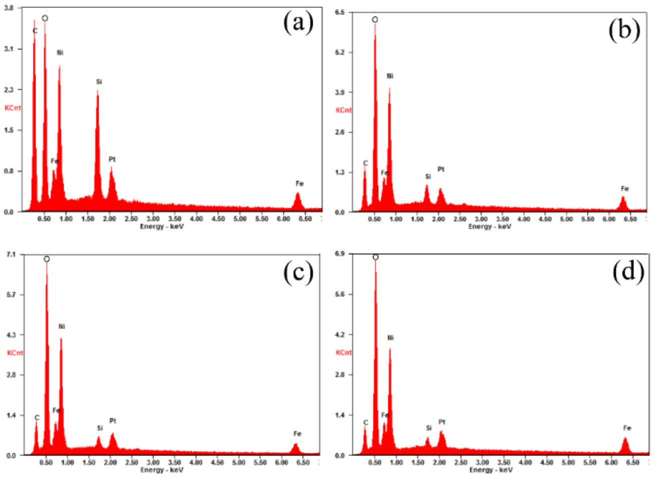

Fig S2 EDS spectrums of (a) LDH-CNFs, (b) LDH-CNTs, (c) LDH-RGO and (d) LDH-RGO-CNFs.

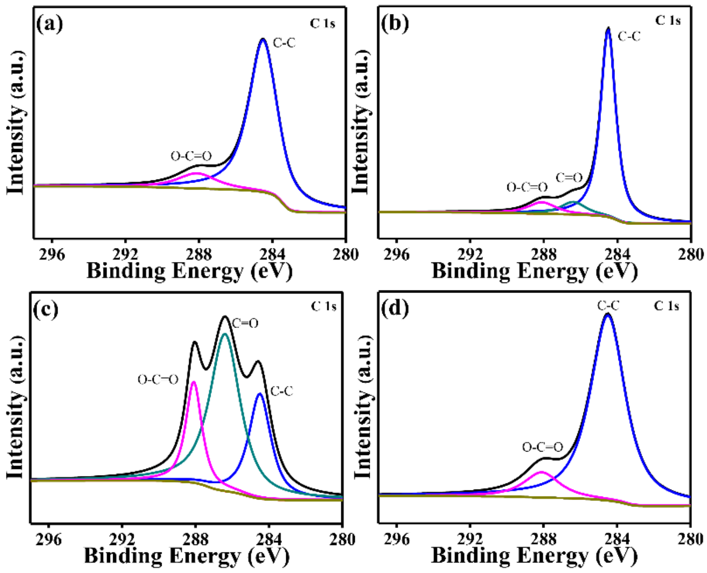

Fig S3 C 1s XPS spectrum of (a) CNFs, (b) CNTs, (c) GO and (d) LDH-RGO-CNFs.

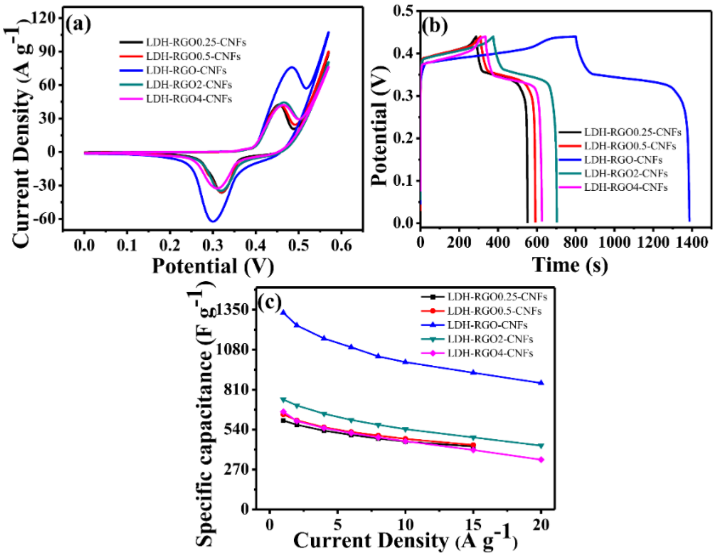

Fig S4 (a) CV curves of the LDH-RGO$_X$-CNFs composites (X= 0.25, 0.5, 1, 2, 4) at a scan rate of 10 mV s^{-1}; (b) GCD curves of the LDH-RGO-CNFs composites (X= 0.25, 0.5, 1, 2, 4) at a current density of 1 A g^{-1}; (c) The specific capacitance of the LDH-RGO-CNFs composites (X= 0.25, 0.5, 1, 2, 4) at different current densities.

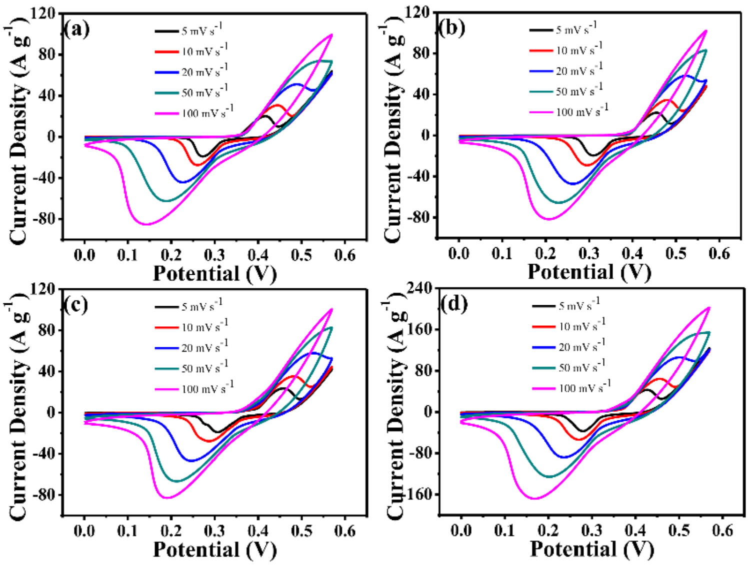

Fig. S5 (a) CV curves of the LDH-CNFs composite at different scan rates. (b) CV curves of the LDH-CNTs composite at different scan rates. (c) CV curves of the LDH-RGO composite at different scan rates. (d) CV curves of the LDH-RGO-CNFs composite at different scan rates.

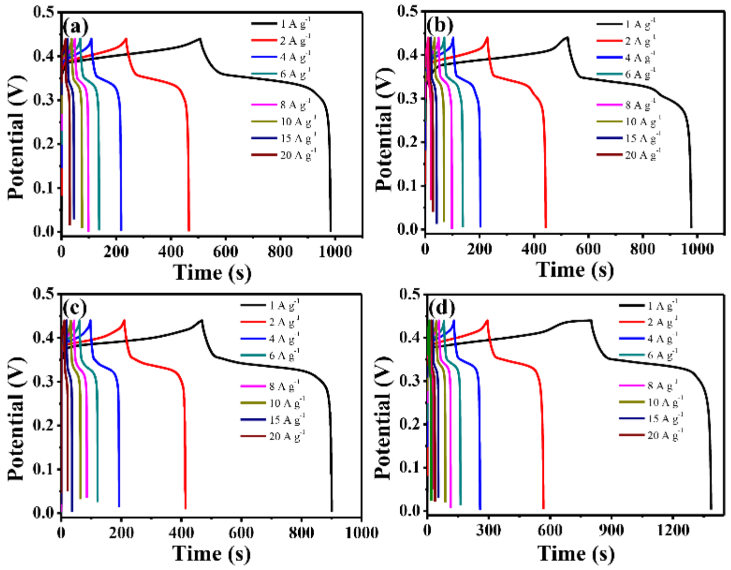

Fig. S6 (a) GCD curves of the LDH-CNFs composite at different current densities. (b) GCD curves of the LDH-CNTs composite at different current densities. (c) GCD of the LDH-RGO composite at different current densities. (d) GCD curves of the LDH-RGO-CNFs composite at different current densities.

Table S1 Specific capacitance of the samples at various current densities.

Current density ($A\ g^{-1}$)	LDH-CNFs ($F\ g^{-1}$)	LDH-CNTs ($F\ g^{-1}$)	LDH-RGO ($F\ g^{-1}$)	LDH-RGO-CNFs ($F\ g^{-1}$)
1	1080.9	1032.3	981.8	1330.2
2	1040.9	975.5	922.7	1244.1
4	989.1	957.3	865.5	1155.5
6	938.2	932.7	818.2	1096.4
8	896.4	892.7	770.9	1034.5
10	856.8	784.1	725.0	995.5
15	780.7	722.7	613.6	923.9
20	690.9	659.1	486.4	854.5
Retention rate	63.9%	63.2%	49.5%	64.2%

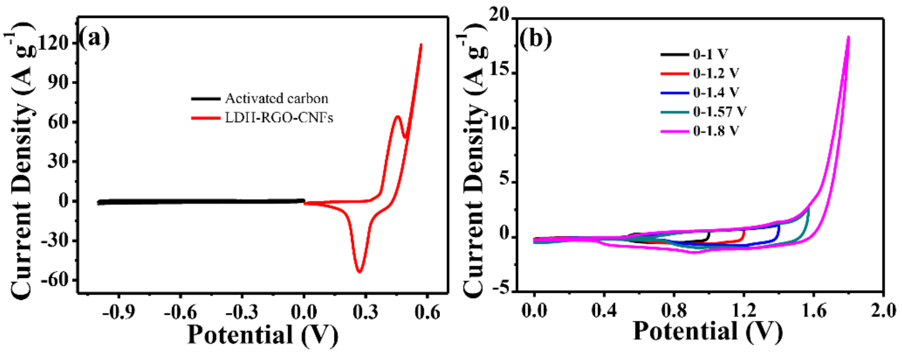

Fig. S7 (a) CV curves of activated carbon and LDH-RGO-CNFs electrodes at scan rate of 10 mV s^{-1}. (b) CV curves at different voltage windows for the ASC at a scan rate of 10 mV s^{-1}.

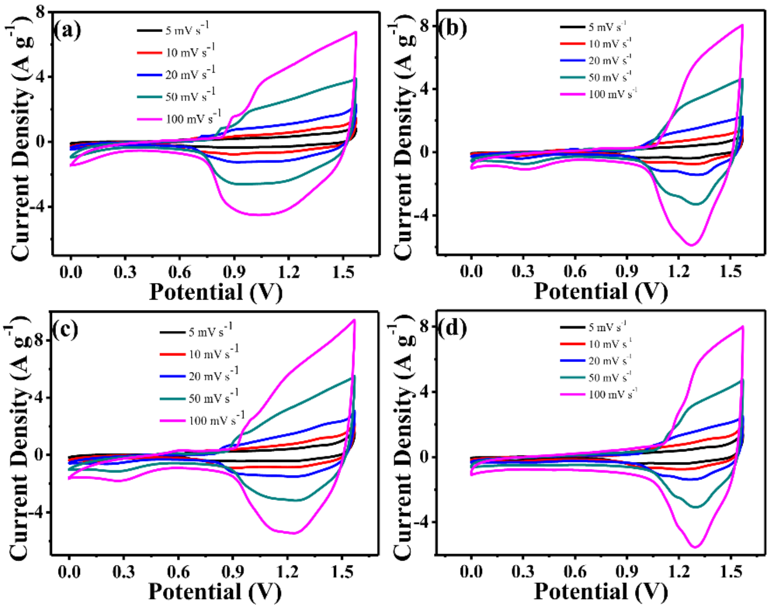

Fig. S8 (a) CV curves of the AC//LDH-CNFs ASC at different scan rates. (b) CV curves of the AC//LDH-CNTs ASC at different scan rates. (c) CV curves of the AC//LDH-RGO ASC at different scan rates. (d) CV curves of the AC//LDH-RGO-CNFs ASC at different scan rates.

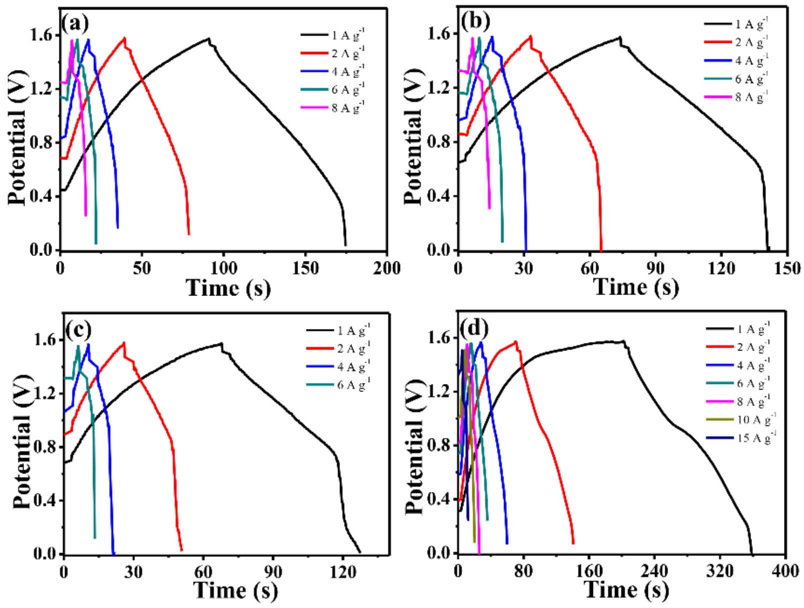

Fig. S9 (a) GCD curves of the AC//LDH-CNFs ASC at different current densities. (b) GCD curves of the AC//LDH-CNTs ASC at different current densities. (c) GCD of the AC//LDH-RGO ASC at different current densities. (d) GCD curves of the AC//LDH-RGO-CNFs ASC at different current densities.

Table S2 Specific capacitance of the ASCs at various current densities.

Current density (A g^{-1})	AC// LDH-CNFs ASC (F g^{-1})	AC// LDH-CNTs ASC (F g^{-1})	AC// LDH-RGO ASC (F g^{-1})	AC// LDH-RGO-CNFs ASC (F g^{-1})
1	53.0	43.0	37.8	98.4
2	49.6	40.4	31.3	87.1
4	44.8	38.5	27.6	75.9
6	41.3	38.1	25	67.9
8	40.2	36.3		65.9
10				65.3
15				61.6
